# Non-invasive Brain Stimulation: A Paradigm Shift in Understanding Brain Oscillations

**DOI:** 10.3389/fnhum.2018.00211

**Published:** 2018-05-25

**Authors:** Johannes Vosskuhl, Daniel Strüber, Christoph S. Herrmann

**Affiliations:** ^1^Experimental Psychology Lab, Center for Excellence “Hearing4all,” European Medical School, University of Oldenburg, Oldenburg, Germany; ^2^Research Center Neurosensory Science, University of Oldenburg, Oldenburg, Germany

**Keywords:** non-invasive brain stimulation, transcranial alternating current stimulation, brain oscillations, causality, mechanisms of tACS

## Abstract

Cognitive neuroscience set out to understand the neural mechanisms underlying cognition. One central question is how oscillatory brain activity relates to cognitive processes. Up to now, most of the evidence supporting this relationship was correlative in nature. This situation changed dramatically with the recent development of non-invasive brain stimulation (NIBS) techniques, which open up new vistas for neuroscience by allowing researchers for the first time to validate their correlational theories by manipulating brain functioning directly. In this review, we focus on transcranial alternating current stimulation (tACS), an electrical brain stimulation method that applies sinusoidal currents to the intact scalp of human individuals to directly interfere with ongoing brain oscillations. We outline how tACS can impact human brain oscillations by employing different levels of observation from non-invasive tACS application in healthy volunteers and intracranial recordings in patients to animal studies demonstrating the effectiveness of alternating electric fields on neurons *in vitro* and *in vivo*. These findings likely translate to humans as comparable effects can be observed in human and animal studies. Neural entrainment and plasticity are suggested to mediate the behavioral effects of tACS. Furthermore, we focus on mechanistic theories about the relationship between certain cognitive functions and specific parameters of brain oscillaitons such as its amplitude, frequency, phase and phase coherence. For each of these parameters we present the current state of testing its functional relevance by means of tACS. Recent developments in the field of tACS are outlined which include the stimulation with physiologically inspired non-sinusoidal waveforms, stimulation protocols which allow for the observation of online-effects, and closed loop applications of tACS.

## Introduction

For more than one century, cognitive neuroscience surged to find the mechanisms behind the functioning of the human brain. Most often, researchers are limited to observe the *result* of brain functioning, i.e., behavior, or record and interpret correlates of brain activity. With the emergence of more precise instruments to measure for example the electrical brain activity and more sophisticated analysis techniques, the insights into brain functioning and the theories about it became more elaborate. For many decades, cognitive neuroscience research mostly followed the same principle of giving a functioning brain a specific task, recording its activity, analyzing this activity and posing a theory of how the observed brain activity relates to the task. Often, conclusions were drawn and theories were developed suggesting a certain feature of the brain (e.g., a brain area, an ERP component, a brain oscillation, or BOLD response, etc.) to be responsible for this specific task or brain function. It has always been a part of the argumentation that the evidence for the theory was correlational in nature. To prove that this correlational evidence is a representation of a causal relationship, researchers have to manipulate the cause and observe the predicted effect. In order to do so, they only had very limited tools at hand. The most prominent tool to show causality between a certain brain structure and a cognitive function was to analyze brain lesions (Karnath et al., 2018). The co-occurrence of a loss of function with a lesion of a specific part of the brain was then interpreted as a causal relationship. While the method seems to convincingly establish causality, it bears many scientific problems (Rorden and Karnath, 2004). First, it is not suitable for studying the function of healthy brains. This poses a problem because lesioned brains may have developed individual strategies to compensate for the defect (Rorden and Karnath, 2004) unless the data is acquired shortly after symptom onset. Second, the groups of patients are often small and inhomogeneous with respect to their lesion and individual anatomy, thus interpretations about functional relevance of a certain part of the brain have to be taken with a grain of salt. Third, most often researchers can only analyze the brain functioning after the lesion, thus comparisons of changes in a cognitive function induced by the lesion are impossible. Even though this traditional neuropsychological approach is still important and can augment current imaging techniques (Rorden and Karnath, 2004), it is not a suitable method to study brain mechanisms on healthy brains in big groups of humans.

**Figure 1:**
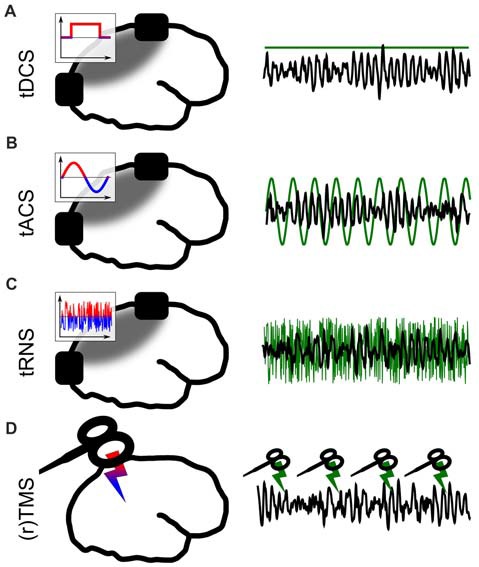
Different forms of non-invasive brain stimulation (NIBS). The left panel depicts a sketch of the respective application. The inlays show the voltage between the electrodes over time. The gray areas depict simplified electric field distributions in which the target area should be located. The right panel represents the stimulation signal (green) relative to EEG (black) from a potential target area.** (A)** Transcranial direct current stimulation (tDCS) via externally attached electrodes. **(B)** Transcranial alternating current stimulation (tACS). **(C)** Transcranial random noise stimulation (tRNS). **(D)** (Repetetive) transcranial magnetic stimulation (TMS).

The solution to this problem came with the development of non-invasive brain stimulation (NIBS; Figure 1). For a long time throughout human cognitive neuroscience, the idea of stimulating the brain by external forces has been present (Guleyupoglu et al., 2013), but it’s use as a tool for research in neuroscience developed only relatively recently (Zaghi et al., 2010). Among others, the most established techniques to stimulate the brain are transcranial magnetic stimulation (TMS; Figure 1D) and transcranial electrical stimulation (TES; Figures 1A–C). In TMS, a strong magnetic field penetrates the skull for a very short duration (<1 ms, Hallett, 2007). This fast change in magnetic field strength induces a current in cortical neurons which is oriented in parallel to the magnetic coil (Hallett, 2000, 2007). TMS allows for very focal and effective stimulation of cortical neurons which elicit action potentials as a result of stimulation (Barker and Shields, 2017). With the second method, TES, a weak electric current is applied to the scalp (Nitsche and Paulus, 2000). A fraction of this current enters the brain and causes a membrane potential change of the affected neurons which is strong enough to change the probability of a neuron generating action potentials (Antal and Herrmann, 2016). This method is generally considered sub-threshold because, in contrast to TMS, no action potentials are directly triggered by the stimulation.

Both TMS and TES are applied in many different forms, which are tailored towards specific research goals. The shape of a single pulse of TMS can be manipulated (mono- or bi-phasic TMS). Magnetic pulses can either be applied as a single pulse, or with pulses being repeated several times at a specific frequency (Hallett, 2007). This repetitive kind of TMS is understood as a method to interfere with ongoing brain oscillations (Thut et al., 2011). TMS can be applied to map cortical functions in space by functionally inhibiting a certain cortical structure to produce a so-called “virtual lesion” (Pascual-Leone et al., 1999) or to excite a structure and measure, for example, TMS-induced muscle contractions (Danner et al., 2008).

TES was first applied using direct current (transcranial direct current stimulation, tDCS; Nitsche and Paulus, 2001) between two electrodes (Figure 1A). It is often used to change cortical excitability with excitatory effects underneath the anode (Fertonani and Miniussi, 2017). In a second form of TES, alternating sinusoidal currents are applied (transcranial alternating current stimulation, tACS; Antal et al., 2008, Figure 1B) between two electrodes. Depending on the frequency, position of the electrodes and strength of the current, different brain oscillations can be targeted and manipulated (Antal and Paulus, 2013; Herrmann et al., 2016b). Thirdly, random noise can be applied (Terney et al., 2008, Figure 1C) to have a general excitatory effect on underlying cortical tissue (Terney et al., 2008). In contrast to tDCS, tRNS does not generally manipulate neuronal excitability. The exact neural mechanisms behind tRNS are not well understood to date, but one likely way of interaction between tRNS and neural activity is suggested to be stochastig resonance. Stochastic resonance describes a process by which sub-threshold brain oscillations become enhanced by adding noise and thus become supra-threshold (see a detailed discussion of neuronal mechanisms in Antal and Herrmann, 2016). Many combinations of the above TES techniques are practiced and within these forms, further specific sub-forms have been developed.

These techniques, which are relatively easy to apply, comparatively cheap and well tolerated by healthy human volunteers, allow the manipulation of brain activity in larger groups of subjects. This holds especially for the TES methods, because TES devices are much cheaper in comparison to TMS and affordable for a wider group of researchers. With these techniques, for the first time, causal relationships could be statistically evaluated and proven between many brain functions and neural signals (Herrmann et al., 2016b; Veniero et al., 2016). Thus, the introduction of NIBS to cognitive neuroscience opens new gates for researchers to understand the neural mechanisms behind cognitive functions.

To understand the effects of NIBS techniques, we have to realize that cognition is formed by the complex interplay of single neurons. Communication between single neurons and computation by neurons is realized by electrical signaling between cells. These electromagnetic fields generated by groups of neurons propagate through the tissue and can be recorded from the scalp during normal brain functioning by EEG or MEG. These electrophysiological signals from the brain are often dominated by rhythmic patterns of different frequencies, the so-called brain oscillations. In EEG/MEG, brain oscillations are traditionally grouped into frequency bands, which were loosely ascribed to different clusters of cognitive functions. To date, thousands of studies investigated the role of brain oscillations for different cognitive functions and it seems to be generally accepted that brain oscillations play a crucial role in brain functioning (Başar, 1998, 1999; Buzsáki, 2006).

Because brain oscillations form one of the most prominent observable features of computation and communication between cells and brain regions in the brain, we will focus our review on tACS, a brain stimulation technique designed to manipulate brain oscillations (Herrmann et al., 2016b).

The neural mechanisms of how NIBS modulates brain activity are still poorly understood. This poses a problem to the argument of causality. When the underlying mechanisms are not known well enough, researchers cannot describe how exactly electric current applied to electrodes on the scalp change the behavioral outcome of a cognitive function. We will thus first review the current state of research on the neural mechanisms behind the effects of tACS. We believe that understanding the mechanisms of action of tACS will also yield considerable insights into brain functioning and human cognition in general. We start by reviewing findings of cognitive and neurophysiological effects of tACS in humans documenting the potential of tACS to affect human cognition. We then draw a line of explanation from animal studies *in vitro* and *in vivo* demonstrating how these effects come about from a neurophysiological perspective. In the second half of this review, we focus on articles using tACS to test established (correlational) theories about the functional relevance of specific features of a brain oscillation, like amplitude, frequency and phase. Finally, we describe some recent technical developments in tACS research and suggest some research topics that might promote the field in the future.

## Alternating Currents on Different Levels of Observation

**Figure 2:**
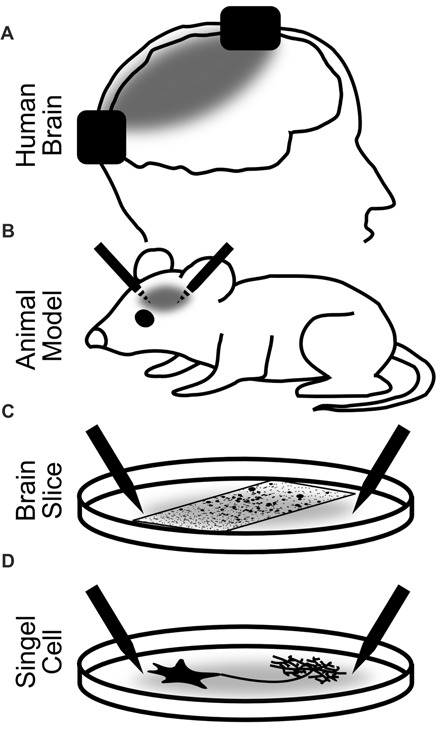
Explanatory levels of brain stimulation with alternating currents. Black rectangles **(A)** and needles **(B–D)** depict the stimulation electrodes. Gray areas illustrate the electric fields between the electrodes. **(A)** Stimulation of the human brain. Most restricted in the insights into the neural dynamics and stimulation parameters but most relevant for the observation of cognitive effects. Most often, non-invasive but also invasive recordings and stimulation have been used. **(B)** Invasive stimulation of animals allows for more precise and controlled stimulation and accurate recordings. Only limited observation of cognitive effects of stimulation is possible. **(C)** Stimulation of brain slices or cell assemblies. Highly controlled stimulation and recording are possible. Network effects of stimulation can be observed but no behavior. **(D)** Stimulation of a single neuron. Observations of the effect of alternating currents on different parts of the neuron down to the single iron channel or synapse are possible.

In humans, the physiological changes of brain activity in response to brain stimulation are mostly measured non-invasively using EEG or MEG. With these methods, however, it is impossible to investigate neuroelectric effects on small groups of neurons. Invasive recordings in animals are therefore pivotal to the field because they allow to directly investigate effects of stimulation on neuronal tissues. In the next sections, we review stimulation effects in humans, which allow for the simultaneous observation of high-level cognitive effects. Then, we will expand our focus to animal studies both *in vivo* and *in vitro*. From these studies, a deeper insight into the physiological effects can be achieved, but the observation of cognitive effects is limited (Figure 2).

### Humans

There is a growing body of literature reporting behavioral effects of NIBS in many areas of brain functioning. Theoretically, any cognitive or other brain function that has been previously attributed to brain oscillations can be investigated with tACS and other NIBS methods. Thus, many higher cognitive functions such as memory (Marshall et al., 2006; Polanía et al., 2012; Vosskuhl et al., 2015), intelligence (Santarnecchi et al., 2013), creativity (Lustenberger et al., 2015), and risk taking (Sela et al., 2012) have been successfully modulated by tACS. This also holds for lower-level cognitive functions like voluntary movement (Pogosyan et al., 2009; Joundi et al., 2012), vision (Strüber et al., 2014; Cecere et al., 2015) and audition (Neuling et al., 2012a). Far more examples have been reviewed recently (Kuo and Nitsche, 2012; Abd Hamid et al., 2015; Veniero et al., 2016). On the basis of these experiments, a causal relationship between function and oscillation is often claimed. Additionally, the wide variety of these findings seem to prove the efficacy of the method. Nevertheless, only a fraction of these studies actually control the mechanism of tACS by recording EEG or MEG. Therefore, even though research using tACS to investigate cognition is a vivid field of research, electrophysiological evidence for its efficacy is still sparse.

#### Non-invasive Recordings

The best way to study the mechanisms of action of tACS and to prove its efficacy, is to observe its effects during stimulation. However, due to the huge electric artifact, which is several magnitudes larger than the physiological EEG signal, such online effects are difficult to obtain. Therefore, many studies report only aftereffects of tACS as the difference between pre and post stimulation measurements. These aftereffects are assumed to be implemented by neural plasticity, while effects during stimulation can be explained by neural entrainment (Pikovsky et al., 2003; Thut et al., 2011). Both mechanisms are further explained in the box “Entrainment and plasticity.” Aftereffects strongly indicate that tACS interferes with cortical neurons and yield important, validating evidence for the efficacy of the method.

Plasticity-induced aftereffects of tACS can relate to different parameters of an internal oscillation. They have been demonstrated in terms of elevated amplitudes at the stimulation frequency (Neuling et al., 2013; Vossen et al., 2015; Kasten et al., 2016), the endogenous frequency (Zaehle et al., 2010; Helfrich et al., 2014b), or in a different frequency band (Neuling et al., 2012a) as well as in terms of prolonged phase-coherence between hemispheres (Helfrich et al., 2014a; Strüber et al., 2014). Note, that entrainment and plasticity are not mutually exclusive and may rely on each other (Vossen et al., 2015).

By definition, entrainment itself does not outlast the stimulation period. Nevertheless, the effect of entrainment does not vanish instantly. For a few cycles after stimulation offset, the internal phase of the oscillation is still coupled to the external force, as has been reported for rTMS stimulation (Hanslmayr et al., 2014) and tACS (Marshall et al., 2006). Notably, entrainment echoes were not always detected after tACS (Vossen et al., 2015), indicating that the phenomenon possibly depends on stimulation intensity and/or duration. Nevertheless, a combination of entrainment and plasticity might explain the observed aftereffects, in that a successful entrainment during stimulation might be a necessary requirement for the generation of synaptic plasticity reflecting enduring aftereffects.

Only recently, the first evidence for the assumed interaction of online entrainment and aftereffect has been reported by Helfrich et al. (2014a,b). These authors were able to demonstrate that the strength of an alpha amplitude increase after stimulation correlated positively with the power during alpha-tACS (Helfrich et al., 2014b). Using gamma-tACS, the same authors reported a correlation between a change in interhemispheric gamma coherence during and after tACS, i.e., the stronger the online modulation strength the stronger the aftereffect (Helfrich et al., 2014a). A direct comparison between online and offline effects in this study was possible due to the broad band activity of gamma oscillations. The authors stimulated at 40 Hz but analyzed online EEG effects at different gamma-subbands. Thus, it was possible to filter out the 40 Hz stimulation artifact. Together, these findings suggest a relationship between entrainment and plasticity, in which stronger entrainment predicts stronger aftereffects.

If entrainment and plasticity were positively related, then a comparable pattern of stimulation frequency and intensity should be detectable. That is, when the stimulation frequency differs from the endogenous frequency, entrainment effects are supposed to be smaller, and in turn plasticity effects should decrease. Indeed, this relationship has been documented in a study applying tACS at individual alpha frequency (IAF) as determined in the first of four sessions (Vossen et al., 2015). This procedure resulted in stimulating closely to the internal frequency in the first session and a little off the internal frequency in some of the remaining sessions, since IAF changes over time. The authors report a slightly larger effect when stimulating just above the internal frequency (Vossen et al., 2015). Even though the study fails to find direct evidence for entrainment (Vossen et al., 2015), the existence of the aftereffect indicates that a secondary mechanism like synaptic plasticity is responsible for the aftereffect to occur.

A different line of research has a focus on the duration of tACS aftereffects. One possible approach in studying aftereffect durations, is to continue EEG-recording for a prolonged time after stimulation offset, but keeping the experimental conditions constant. Using this approach, Kasten et al. (2016) demonstrated an alpha tACS-induced amplitude enhancement which outlasted the stimulation by up to 70 min. Notably, this effect did not vanish due to a return to baseline, but because of a steady fatigue-related increase of the resting state alpha amplitude in the sham-stimulated group. Thus, the plastic changes due to tACS might persist for even longer time periods (Kasten et al., 2016). This result together with other studies (Neuling et al., 2013) indicates that the pre stimulation alpha amplitude is an important parameter when aftereffects are observed. Therefore, relevant context effects like ambient illumination levels, that are known to affect alpha amplitude, have to be taken into account when designing alpha modulation studies (Stecher et al., 2017).

#### Intracranial Recordings

Recording electric brain signals directly from the surface of the brain bears many advantages in comparison to scalp-recorded EEG or MEG. Most importantly, the signal quality is much better due to the lack of dampening effects of skull and other tissues. However, a major limitation of this technique is its restriction to case studies of patients, mostly suffering from epilepsy, with electrode grids implanted for the pre-operative localization of epileptic foci. This condition prevents researchers from choosing recording sites freely. The method itself, however, promises substantial insights into the mechanisms of AC-stimulation, especially if the stimulation is applied via the same electrode grid, leading to an enormous increase in focality compared to transcranial application.

So far, there are only very few studies combining intracranial recordings with AC stimulation (Alagapan et al., 2016; Opitz et al., 2016; Peterchev, 2017) with only one measuring oscillatory brain activity in response to AC stimulation (Alagapan et al., 2016). In this study, subdural electrode grids over parietal areas were used for recording and stimulating three epilepsy patients. Stimulation was applied as biphasic pulses (square waves) at 10 Hz for periods of 5 s. This rather unconventional stimulation signal led to results resembling those of sine wave tACS (Neuling et al., 2013). In a state of high endogenous alpha amplitudes, i.e with eyes closed, the entrainment effect was weak, whereas in a state of low alpha amplitudes, with eyes open, a stronger amplitude was detected together with a shift of the internal frequency towards the stimulation frequency (Alagapan et al., 2016). The effects at least outlasted the stimulation for the analyzed period of 5 s, which cannot be attributed to plasticity effects, because 5 s of stimulation is considered too short to induce plasticity (Strüber et al., 2015; Vossen et al., 2015). Thus, the effects are interpreted as consequence of entrainment (Alagapan et al., 2016). These results, however, should be interpreted carefully, as the experimental set-up differs markedly from standard tACS protocols in many respects. First, a square wave might cause different effects than a sinusoidal wave, since it has been argued that waveform shape represents an important feature of brain oscillations (Cole and Voytek, 2017). Modifying the waveform shape of alpha tACS for example changed the effectiveness of stimulation (Dowsett and Herrmann, 2016). The particular form of a brain oscillation is starting to be acknowledged in the tACS literature only recently. Current approaches are discussed under “Recent Developments—Stimulating With Complex Waveforms” section. Second, the authors applied a stimulation intensity of 2 mA directly to the brain which is considerably stronger than the typical application of 1–2 mA to the scalp. Third, results rely on only three patients with electrodes implanted over roughly overlapping sites, thus spreading the location of stimulation and recording quite considerably.

The other two studies using AC stimulation and intracranial recordings didn’t analyze their data with respect to brain oscillations, but provide important information about the electric field strength measured inside the skull while AC fields are applied at the scalp (Opitz et al., 2016; Huang et al., 2017). For a typical stimulation intensity of 1 mA, Opitz et al. (2016) reported electric fields in the range of 0.1–0.3 V/m. For the same stimulation intensity, Huang et al. (2017) measured electric fields up to 0.2 V/m. Another notable article tested intracranial field strengths when tDCS is applied to patients with implanted deep electrodes (Chhatbar et al., 2018). The authors measured electric fields of 0.1 V/m in subcortical areas for 2 mA tDCS, indicating stronger fields in cortical areas, which probably correspond to values measured under tACS. These articles thus confirmed previous models of electric fields, which were based solely on brain models derived from MR images (Neuling et al., 2012b; Datta et al., 2013). These finite element models predicted electric field strengths at cortical target areas in the range of 0.3 V/m (Antal and Herrmann, 2016), a field strength that has been demonstrated in animal experiments to be strong enough to influence the firing pattern of neurons (Antal and Herrmann, 2016).

### Behaving Animals

A very interesting example of a successful translation of animal findings to humans is represented by a set of studies where a highly similar stimulation protocol is applied first to humans and then to macaque monkeys (Kar and Krekelberg, 2014; Kar et al., 2017). In human participants, the authors found a change in motion perception that was attributed to a modulation of neural plasticity induced by tACS (Kar and Krekelberg, 2014). More precisely, the stimulation led to a change in the strength of motion adaptation. When visual stimuli move into a coherent direction for a prolonged amount of time, the visual system adapts to the movement and subsequently perceives movement in the opposite direction when the movement of the visual stimulus is stopped. The strength of that effect was weaker after the application of tACS. In the experiment on macaque monkeys, Kar et al. (2017) recorded local field potentials (LFPs) directly from the visual area MT during 10 Hz tACS with 2 mA, applied to the scalp of the monkeys while the monkeys observed moving visual stimuli. As in the human data, the authors found a tACS-related attenuation of movement adaptation. In the macaque data, however, the authors could directly demonstrate a change in the firing pattern of motion sensitive neurons, indicating a modulation of neural plasticity which is thought to underlie the natural motion after effect (Kar and Krekelberg, 2014; Kar et al., 2017).

In addition, their recording setup allowed for a measurement of the electric field strength induced by tACS (Kar et al., 2017). The lateralized stimulation generated an electric field of 0.16 V/m as measured in area MT in the stimulated hemisphere, whereas in the other hemisphere the electric field reached only 0.03 V/m. These measurements demonstrate that their tACS setup induced electric fields that are strong enough to manipulate neural activity as has been assumed earlier (Ozen et al., 2010; Antal and Herrmann, 2016).

Inspired by early reports of tACS-effects on human cognition (Marshall et al., 2006), Ozen et al. (2010) used electrodes implanted on the surface of the skull of anesthetized rats to apply alternating current at low frequencies (0.8–1.7 Hz). LFPs, multi- and single-unit activity were measured via additionally implanted electrodes directly from the brain. Under anesthesia, rats produced a slow oscillation below 2 Hz (Ozen et al., 2010), thus an endogenous oscillation was established. Such an endogenous oscillation is considered a prerequisite for entrainment (see Box 1—*“Entrainment and Plasticity”*). Externally applied AC electric fields successfully changed the firing pattern of single cells in both neocortical and hippocampal areas. The stronger the electric field, the more cells adapted their firing patterns. In the awake rat, when the animal did not show a slow endogenous oscillation, the same stimulation resulted in a much weaker effect. The authors interpreted this effect as frequency-specificity of AC stimulation (Ozen et al., 2010). With the concept of the Arnold Tongue in mind, these data can be explained in the framework of entrainment.

Box 1 **Entrainment and Plasticity.**

The term entrainment denotes a phenomenon occurring in many different natural systems (Pikovsky et al., 2003). In general terms, entrainment means that the rhythmic activity of two systems is adapted to one another or synchronize with each other. One of the systems is a neural oscillator in the brain, whose oscillatory features are changeable. The other system in case of neural entrainment is an external rhythmic driving force, such as an alternating current, magnetic pulses or flickering lights, tones, etc. An influential list of features that an oscillation must cover in order to qualify as “neural entrainment” has been formulated by Thut et al. (2011):

1. A natural oscillation must be present or at least possible under natural conditions in the range of the frequency of the external rhythmic force.
2. A periodic external force is applied. It can be electric, magnetic, visual, auditory, etc. and its shape can be sinusoidal, a square-wave, repeated pulses, or any other periodic signal.
3. The internal oscillation must synchronize to the external force, i.e., its phase and frequency adapt to the external signal.
4. The effect must be direct, i.e., there should be no secondary stages, such as connected brain areas or other frequencies that mediate the effect.

Point one is crucial in many research applications of entrainment and illustrates the basic difference between rhythmic (tACS, rTMS) and other stimulation techniques (tDCS). If entrainment effects are expected, the researcher surges to manipulate rhythmic brain activity, not the basic excitability of the cortex. This includes the natural occurrence of an oscillation at the stimulation frequency. If before stimulation, the neural tissue does not exhibit rhythmic activity in the respective frequency, or if such an activity is not physiologically possible, entrainment can not be effective. The concept can be extended to also hold for (sub-) harmonics of the stimulation frequency. Additionally, in *in vitro* experiments pre-stimulaiton oscillations have to be induced by chemicals thus not representing strictly natural conditions.

A further phenomenon observed in systems during entrainment is the following: The greater the difference between the internal and the external frequency the stronger is the force needed by an external rhythm to entrain an internal oscillation (Herrmann et al., 2016a; Thut et al., 2017). This phenomenon is referred to as the “Arnold Tongue” (Pikovsky et al., 2003, Figure 3). The presence of an Arnold Tongue in brain stimulation data has been suggested as an indicator of entrainment in human EEG data (Antal and Herrmann, 2016; Notbohm et al., 2016). Thus, if an Arnold Tongue is detected in a stimulation experiment, this is a strong indicator for entrainment.

Entrainment effects can outlast stimulation offset by a few cycles at most (Halbleib et al., 2012; Hanslmayr et al., 2014), thus longer lasting electrophysiological effects can not be explained in the framework of entrainment (Chaieb et al., 2011; Strüber et al., 2015). Such effects have been hypothesized to rely on neural plasticity as a possible mechanism (Zaehle et al., 2010; Polanía et al., 2012; Vossen et al., 2015)—especially “long-term-potentiation” (LTP) and “long-term-depression” (LTD), which are elicited by spike-timing-dependent plasticity (STDP, Markram et al., 1997; Zaehle et al., 2010). Under natural conditions, the mechanism is the following: If an action potential arrives at a synapse shortly before a post-synaptic potential change, the respective connection will be strengthened (LTP). If, however, an action potential arrives shortly after a post-synaptic potential change, the connection will be weakened (LTD). During tACS, the alternating electric field repetitively depolarizes and hyperpolarizes the cell membrane, probably resulting in STDP. A more detailed explanation of this idea can be found elsewhere (Zaehle et al., 2010).

**Figure 3:**
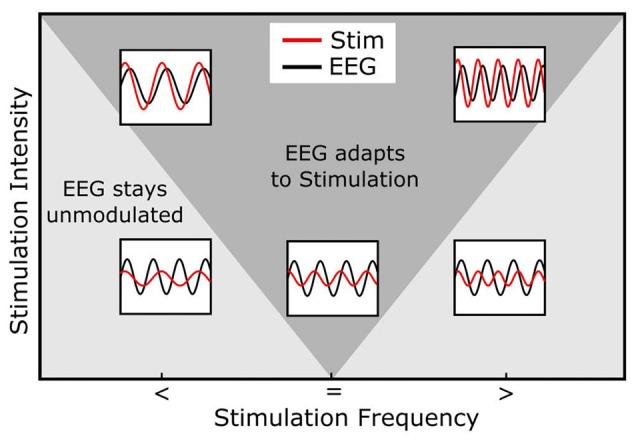
Arnold Tongue. The horizontal axis represents the stimulation frequency with smaller (<), equal (=) or higher (>) stimulation frequencies relative to the endogenous frequency. The vertical axis shows the stimulation intensity. The inlays depict the relationship between the endogenous (e.g., EEG, black) and stimulation signal (red). Note how the stimulation signal increases in amplitude from bottom to top and how its frequency relates to the EEG signal. During stimulation, the endogenous oscillation couples in phase and frequency to the stimulation signal (entrainment) in cases inside of the Arnold Tongue (dark gray area) and stays unmodulated out of the Arnold Tongue (light gray area). When the stimulation frequency is identical with the endogenous frequency, even low stimulation intensities lead to a coupling of the two oscillations (central, lower inlay). The same intensity does not lead to entrainment, when the stimulation frequency is not matched to the endogenous frequency (lower left and right inlays). If, however, the intensity is increased, these non-matched frequencies do lead to entrainment (upper left and right inlays).

An experiment with anesthetized ferrets confirmed these results (Ali et al., 2013), extending the finding to another animal model and to a variety of different stimulation protocols. Ferret brains, in comparison to rat brains, are bigger and show a higher degree of gyrification, thus representing a more complex model organism. Ali et al. (2013), who also published a computational model of tACS effects in the same article, were able to demonstrate that an external AC field interferes with an endogenous oscillation, depending on its frequency relative to the endogenous frequency and its intensity (Ali et al., 2013).

### Brain Slices and Single Cells

Slice preparations and observations on single cells *in vitro* provide an even higher level of control over recordings and stimulation. At this level of observation, the electric field strength can be set very accurately to manipulate the observed cells, membrane potentials can be measured, and even endogenous oscillations can be designed using specific chemicals. Thus, to understand the mechanisms that constitute cognitive effects of tACS, it is necessary to prove that the application of alternating current has an effect on single cells and on cell assemblies.

To understand effects of alternating fields, it might be useful to start with constant electric fields and their impact on cell membranes and firing patterns. When a pyramidal cell is exposed to a constant external electric field, its firing rate is manipulated systematically (Gartside, 1968; Bikson et al., 2004). The exact location on the cell membrane where the polarization shows its effects has been investigated (Bikson et al., 2004; Rahman et al., 2013; Chakraborty et al., 2017). Research revealed that certain parts of the neuron are more susceptible to polarization than others (Chakraborty et al., 2017) and that dendrites and cell bodies are usually oppositely charged in a radial electric field (Rahman et al., 2013; Philip et al., 2017). Thus, on single-cell level the effects of cathodal and anodal currents may be complex (Bikson et al., 2004) and are dependent on the orientation of the cell relative to the electric field gradient. However, this level of complexity exceeds the scope of the explanatory model of brain stimulation effects applied in this review article. We will adapt a more functional level to understand the effects of current stimulation.

We refer to a cell as positively polarized when it increases its firing rate due to an electric field applied to it. A negatively polarized cell is considered one that decreases its firing rate (Gartside, 1968; Antal and Herrmann, 2016). Positive voltage leads to a depolarization of the cell membrane, thus shifting the membrane potential closer to the firing threshold and, therefore, the cell will elicit more action potentials compared to the non-stimulated case. Under negative voltage, this effect is reversed (Chakraborty et al., 2017). Thus, it is evident that static electric fields directly manipulate cell excitability and, thereby, have the potential to change neuronal processing.

One important problem in this area of translational research is the lower boundary of the stimulation intensity for the manipulation of neuronal activity. In the literature, there are generally two different positions on what it means to “manipulate neuronal activity.” The first position is that only an increase or decrease of firing rates constitutes a manipulation of neuronal activity (Vöröslakos et al., 2018). The second position states that also a change in the timing of neuronal firing is a sign of manipulated activity, without necessarily producing a net change of firing rates (Fröhlich and McCormick, 2010). Although the first interpretation nicely fits the mechanistic explanation of direct current effects, it misses possible effects of the defining characteristic of alternating current stimulation, i.e., the rhythmic change of the electric field polarity. The rhythmic change of electric field polarity in turn, leads to a change in the temporal firing patterns of neuronal tissue (Fröhlich and McCormick, 2010). In the following, we will thus interpret a change of the temporal pattern of neuronal firing as a successful manipulation of neuronal activity in the context of AC stimulation.

The theory of entrainment suggests that theoretically any field strength can induce entrainment, given an exact match of stimulated and manipulated frequencies. In practice, however, there is probably a lower limit which is investigated *in vitro*. Fröhlich and McCormick (2010) found different lower boundaries for the manipulation of neuronal activity when stimulating with a sine wave compared to a physiological signal. With a sine wave, approximately matched in frequency with the endogenous oscillation in a ferret hippocampal slice, a significant effect is reported for a field strength of 1 V/m (Fröhlich and McCormick, 2010). For 0.5 V/m, the lowest tested field strength in the article, the authors report a *p*-value of 0.16. Given their relatively low number of observations (*N* ≤ 10) and that the stimulation frequency was not precisely matched, a field strength of 1 V/m might not form the absolute lowest boundary. When stimulation is applied as a physiological wave-form (pre-recorded signal) a significant modulation of the endogenous oscillation is reported at 0.5 V/m but not at 0.25 V/m. Thus, a lower boundary between 0.25 and 0.5 V/m is suggested by the authors. This lower boundary is further substantiated by a simulation of neuronal entrainment by Reato et al. (2010), who reported a strong effect for coherence (*p* < 0.001) between endogenous and stimulation signal at their lowest tested intensity of 0.2 V/m when a sinusoidal field was applied at the endogenous frequency. This result suggests a boundary even below 0.2 V/m if frequencies are precisely matched. A further discussion of the issue of electric field intensities and can be found in Antal and Herrmann (2016).

A very influential and recent publication suggested stimulation intensities much higher than the standard of <2 mA to induce TES effects (Vöröslakos et al., 2018) and thereby triggered a vivid discussion even in advance of its final publication (Underwood, 2016; Ruhnau et al., 2018). Vöröslakos et al. (2018) applied TES to rats, while measuring firing rates of neurons and electric field strengths induced by the applied currents. From these data, they conclude that electric fields of 1 V/m are necessary to significantly alter firing rates of neurons in the intact rat brain. They also measured and estimated that in humans a stimulation intensity of 4–6 mA is required to reach that field strength. Clearly, the lower boundary found in this work is much higher than the previously discussed values (<0.2 V/m) and with this presupposition in mind, many of the already published and replicated experiments from the field of TES would be deemed inexplicable, since the standard stimulation intensity in TES experiments on humans is below 2 mA. We would like to explicitely point out here that the threshold of 1 V/m is relevant only for the manipulation of firing rates, irrespective of ongoing activity. In tACS studies however, the mechanism of action is not an instantaneous change in firing rates of the stimulated tissue, but a manipulation of temporal firing patters. Vöröslakos et al. (2018) acknowledged this difference and interpreted endogenous activity as an impeding factor for their stimulation. Thus their article is only indirectly related and definitely not opposing the TES literature.

It is important to note here that the fields applied in *in vitro* studies are subthreshold, i.e., they do not directly induce action potentials (Fröhlich and McCormick, 2010). In fact, the fields applied in previous *in vitro* experiments are comparable in strength to the fields reaching the cortex in human TES setups (Antal and Herrmann, 2016) as has been discussed above (see section “*Intracranial Recordings*”). Therefore TES must be considered a class of sub-threshold stimulation techniques.

If the external field changes polarity rhythmically as in the case of alternating current stimulation the cell will cycle from positive to negative polarization in the same frequency as the external current. This should lead to a rhythmic increase and decrease in firing rates relative to a natural state. In a setup comparable to that used for direct current stimulation *in vitro* also alternating currents have been tested on single cells and brain slices (Deans et al., 2007; Fröhlich and McCormick, 2010).

In order to investigate entrainment effects of current stimulation, the stimulated neuronal tissue must show a self-sustained oscillation which can then be modulated by alternating currents (see Box 1—*“Entrainment and Plasticity”*). *In vitro*, kainic acid induces activity in the beta/gamma range (15–100 Hz; Buhl et al., 1998) when applied to hippocampal slices for example. These endogenous oscillations serve as a baseline activity to form a neural oscillator.

In experiments on rat hippocampal slices, such a neural oscillator was put under the influence of an alternating current electric field (Deans et al., 2007). In their study, Deans et al. (2007) reported the effect of different frequencies and current strengths of the electric field applied and, thereby, mapped the Arnold Tongue. When applying a 50 Hz alternating current field to a 30 Hz oscillating slice, they found that the frequency of the neural oscillator changed to 25 Hz, a sub-harmonic of 50 Hz. In this scenario, every second cycle of the external electric field triggered one cycle of the internal oscillation. Within the framework of entrainment, this observation constitutes an Arnold Tongue at a sub-harmonic frequency (Antal and Herrmann, 2016). Therefore, the effect was interpreted as an indicator of neural entrainment (Deans et al., 2007). By applying different field strengths, the authors found a minimal current strength, which was necessary to entrain the chemically induced oscillation. The field strengths which showed an effect in their experiments (0.5 V/m) were well beyond the strength of naturally occurring oscillatory field strengths in the rat hippocampus (8 V/m) and therefore within the limits of natural oscillations.

In a highly sophisticated *in vitro* experiment, Schmidt et al. (2014) induced an oscillation to a neocortical slice of mouse brain via optogenetic stimulation. Thereby the parameters of the oscillation were fixed at a determined frequency and phase. Simultaneously, they applied alternating current fields to the tissue at different frequencies and intensities. This procedure enabled the authors to sample different endogenous frequencies with different combinations of exogenous frequencies and amplitudes. With this experimental design, the article reports data for different stimulation frequencies relative to endogenous frequency, different endogenous frequencies and different stimulation strength. Their results confirmed a dependency of the susceptibility of a neural network to adapt to the external electric field depending on the electric field’s frequency and amplitude, thus forming an Arnold Tongue around the (externally controlled) internal oscillation.

## Testing Correlational Theories With tACS

Based on the abovementioned evidence of the efficacy of tACS and its mechanisms, we will now turn towards the impact of tACS for testing long-standing theories about the functional role of brain oscillations in cognition, which we regard as a potential paradigm shift in neuroscience.

Brain oscillations are characterized by different features which have been attributed to different roles in cognition. By far the most prominent feature of an oscillation is its frequency. Brain oscillations have been categorized into frequency bands from their discovery (Berger, 1929). In addition to frequency, other features of a brain oscillation are its phase, amplitude, its specific form, location, its stability relative to other oscillations or locations, and so on. Most of these features have been linked to cognitive functions at different levels and with varying degrees of specificity and mechanistic plausibility (see below for more details).

**Figure 4:**
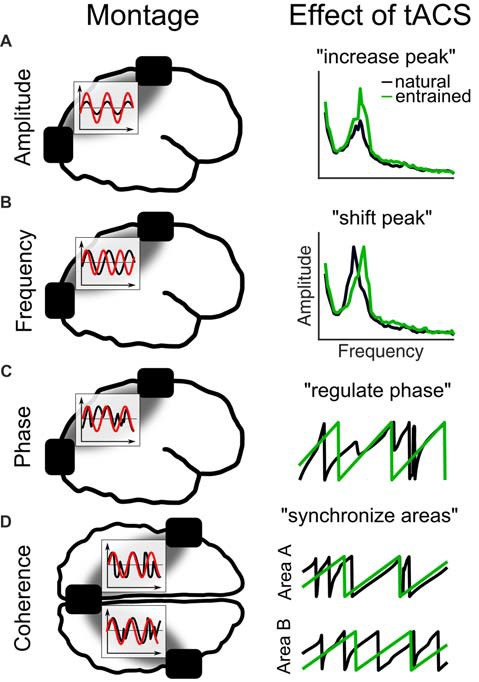
Different tACS designs and their effects on EEG measures. The left panel shows the electrode montage, simplified electric fields between electrodes (gray) and inlays with the stimulation signal (red) relative to the EEG (black). **(A)** External current is applied at the natural frequency. As a result in the frequency spectrum, the amplitude of the natural frequency (black) increases due to entrainment (green). **(B)** Alternating currents is applied at a frequency slightly above the natural frequency. The effect is a shift of the natural frequency to the tACS frequency. **(C)** Current is applied at the natural frequency. The phase of the natural frequency synchronizes to the phase of tACS and is more regular during stimulation. **(D)** When three electrodes are used, one can be connected to one pole and the other two to the other pole of the stimulation device. Thereby, the phase underneath the coupled electrodes is the same and areas underneath will synchronize their endogenous phases to the tACS.

To study the causal role of each of these features in isolation using tACS is challenging, because a natural consequence of entrainment is that several parameters of the oscillation are manipulated at once. Therefore, different tACS setups and experimental designs are necessary to manipulate for example an oscillation’s frequency compared to its phase (Figure 4). For example, when the amplitude of an oscillation is to be enhanced (Figure 4A), also its frequency will be shifted (Figure 4B) or at least stabilized and its phase will be regulated (Figure 4C). When two electrodes are applied over both hemispheres also the phase coherence between stimulated areas might be altered (Figure 4D). Thus, testing a specific parameter requires to carefully design the experiments and to take into account “side effects” on other parameters as an unavoidable result of the stimulation setup.

In the following, we review EEG and MEG studies focusing on one of the different parameters of an oscillation separately together with the respective tACS studies. The studies are sorted according to amplitude, frequency, phase and coherence effects of their most important findings, even though several studies include results about the other parameters as well.

### Amplitude

Alpha waves are clearly visible in EEG recordings over occipital areas due to their immense power. The amplitude of a brain oscillation is a major charachteristic of a brain oscillation. In the context of visual perception, the amplitude of alpha oscillations has been highlighted as a decisive factor of attention. During periods of high alpha amplitude, stimuli are less probable to be detected as compared to states of low alpha amplitudes (Ergenoglu et al., 2004; van Dijk et al., 2008). In accordance with these findings, alpha oscillations have been proposed to reflect states of cortical inhibition as a result of top-down regulation (Klimesch et al., 2007; Jensen and Mazaheri, 2010). The “Gating-by-Inhibition” theory (Jensen and Mazaheri, 2010) proposes that higher order cortical areas functionally inhibit task-irrelevant, e.g., sensory, areas by up-regulating the alpha-activity in the respective cortical area. As a result less sensory input is processed.

Due to its highly mechanistic approach, this theory of alpha amplitude is well suited to be tested by tACS. However, we are not aware of any study directly testing the predicted relationship of increased alpha amplitude and decreased detection rates of visual stimuli following alpha tACS. One notable finding pointing towards this relationship is an experiment using rTMS over the visual cortices at different frequencies. The authors were able to demonstrate that rTMS in the alpha range, but not in the theta or beta range, suppressed detection for stimuli presented contralaterally to the site of stimulation (Romei et al., 2010). This is in line with the proposed gating-by-inhibition model which predicts decreased detection rates for stimuli presented during periods of high alpha amplitudes (Jensen and Mazaheri, 2010).

The presentation of visual stimuli results in an amplitude reduction of the occipital alpha oscillation for a short period after the event (Pfurtscheller and Lopes da Silva, 1999), a phenomenon termed “Event Related Desynchronization” (ERD; Pfurtscheller and Lopes da Silva, 1999). ERD has also been observed during complex cognitive operations, such as mental rotation (Michel et al., 1994) and memory (van Winsun et al., 1984; Dujardin et al., 1995). Generally, more complex cognitive operations result in stronger ERD. Also cognitive performance was observed to improve when ERD was increased (Zoefel et al., 2011).

In this context, the causal relationship between the strength of ERD and the performance in mental rotation was investigated in a recent study (Kasten and Herrmann, 2017). tACS at the IAF was administered during a mental rotation task. The performance on this task was increased relative to a pre-stimulation baseline after tACS, with a sustained aftereffect of at least 55 min (Kasten and Herrmann, 2017). This behavioral effect was accompanied by an increase in ERD. Contrary to their initial hypothesis, the authors also found an increase in task performance during stimulation. A detailed discussion of this unexpected finding can be found in Kasten and Herrmann (2017). All of these findings support the notion of a causal relationship between ERD strength and cognitive performance. The authors hypothesized that the enlarged ERD might be mostly driven by an increase in the alpha-power before stimulus presentation, rather than by a decrease in power after stimulus presentation.

### Frequency

The frequency of a brain oscillation within a specific frequency band has been assumed to be relevant for many cognitive functions. Here, we refer to some examples illustrating the role of frequency of an oscillation for perceptual, mostly visual processes and working memory. With this selection we want to demonstrate the ubiquity of brain oscillations in cognitive processes.

Evidence for a role of the alpha frequency in perception stems from research on the sound-induced flash-illusion (Shams et al., 2002, 2005). When a single visual flash is accompanied by two auditory beeps within a certain window of time, then the single flash is sometimes perceived as a double flash (Shams et al., 2005). Interestingly, the critical time window seemed to be determined by the frequency of the alpha rhythm. When one beep was presented simultaneously to the flash and the other beep was presented less than 100 ms later or earlier, i.e., within the same cycle of an alpha rhythm, the illusion set in (Shams et al., 2002; Experiment 2). Participants with a lower alpha peak frequency showed a longer window for the illusion, presumably because of the longer cycle length at lower frequencies (Cecere et al., 2015). Additional evidence for the relevance of the alpha frequency in the visual system stems from a purely visual phenomenon (Samaha and Postle, 2015). When two short (40 ms) flashes of light are presented with blanc periods of varying duration, these two flashes were occasionally perceived as one. Interestingly, the minimum gap duration at which the participants were able to discriminate the two flashes was dependent on their individual alpha peak frequency (Samaha and Postle, 2015), that is, the faster the alpha frequency the better the temporal resolution.

Causal evidence for the temporal resolution of the visual system to depend on the individual peak frequency of alpha was given by stimulating participants either slightly below or above their individual frequency and determining the length of the window to induce the sound-induced flash-illusion (Cecere et al., 2015). This setup was designed to change the frequency of the internal alpha oscillation and in turn to shorten cycles for tACS above the IAF and elongated cycles for tACS below the IAF. The change of the cycle length should result in a prolonged or shortened window to induce the sound-induced flash-illusion. As expected, the authors found a shrinking of the temporal window of the illusion when stimulating above and an increase in the window duration when stimulating below the IAF (Cecere et al., 2015). Thus, they elegantly tested the mechanistic account of the sound-induced flash-illusion using a psychophysical experiment. Unfortunately, the authors did not record EEG or MEG to demonstrate the assumed change of alpha frequency on the neurophysiological level.

Another visual illusion which is related to the IAF, has been tested recently (Minami and Amano, 2017). This article provides behavioral as well as neurophysiological (MEG) data to control the effects of tACS. The motion-induced spatial conflict (Arnold and Johnston, 2003) describes a specific phenomenon in the context of moving visual stimuli. The combination of two moving visual stimuli induces the illusion of a jittered movement.

The frequency of the jitter presumably lies within the alpha band (Amano et al., 2008; Minami and Amano, 2017). On the basis of this finding, Minami and Amano (2017) applied tACS below and above the IAF while recording MEG data. The stimulation signal was a high-frequency sine wave, amplitude-modulated at the target frequency in the alpha range. This procedure allows to analyze MEG data during stimulation (Witkowski et al., 2016). The rationale behind this approach is further elaborated in section “*Recent Developments—Artifact Reduction*.” Minami and Amano did not only find a highly significant correlation of the individual jitter frequency with the alpha frequency before stimulation, but also demonstrated a change of the perceived jitter frequency due to tACS. The authors also reported a change of the endogenous alpha frequency in the direction of the tACS during stimulation (Minami and Amano, 2017).

These studies on perceptual illusions show that the specific phenomena can be manipulated by tACS in a specific frequency. These experiments also add insights to the notion that alpha may serve as a clock for perception. An idea reviving the old question whether perception is discrete or continuous (VanRullen and Koch, 2003). This hypothesis describes the alpha oscillation in perceptual cortices as an interleaved series of states of high and low excitability realized by peaks and troughs of the alpha wave respectively. In states of low excitability (down-states), less sensory information is processed compared to states of high excitability (up-states). Within this theory, perception is not continuous but discrete at the frequency resembling the IAF. That scenario is corroborated by the studies reviewed above. Overall, the studies reviewed in this section add causal evidence to the relationship between rhythmic activity in the alpha range measured from the visual cortex and functional rhythmicity in visual perception. Notably, in the auditory cortex, gamma—rather than alpha—seems to represent as the frequency of perceptual resolution (Baltus and Herrmann, 2015; Baltus et al., 2018).

The theta band (3–8 Hz) as measured over fronto-medial areas is associated with higher cognitive functions, for example executive functions (Luu et al., 2004; Cavanagh et al., 2012). One elaborate theory on the functional role of theta frequency is the theta-gamma coding model of short-term memory capacity (Lisman and Idiart, 1995; Jensen and Lisman, 1998). Sternberg (1966) made the observation that the reaction time to a memory task increased with a higher memory load. Most interesting about this phenomenon was the stepwise increase by roughly 35 ms per additional item, which represents exactly the cycle length of a gamma-band oscillation (30–80 Hz). More than 30 years later, this finding was integrated into a short-term memory model which suggests the maximal capacity of short-term memory to be determined by the ratio of the individual gamma to theta frequency ratio (Jensen and Lisman, 1998). Theta is the guiding rhythm which is thought to initiate an updating process of the single memory items 3–8 times per second, where the memory items are represented by cycles of a gamma oscillation (Jensen and Lisman, 1998). Thus, if a person has an individual theta frequency of 7 Hz and a gamma frequency of 42 Hz, the model predicts a short-term memory capacity of six items (42/7).

This model may in principle rely solely on the fact that numerically the gamma and theta ratio resembles a value in the range of 7 ± 2, the “magical number” of short-term memory capacity (Miller, 1994). Correlational support for this theory has been accumulated (Siegel et al., 2009; Axmacher et al., 2010; Kamiński et al., 2011), but only by probing this theory with brain stimulation, this relationship can be established as causal. Testing this idea, Vosskuhl et al. (2015) manipulated the capacity of short-term memory by increasing the cycle length of the theta oscillation. Thus, if the respective gamma rhythm remains unchanged, more gamma-cycles fit within one theta-cycle and, in turn, more items can be held in short-term memory. tACS was administered at a frequency slightly below the individual theta frequency which led to an increase in short-term memory capacity in the stimulated, relative to a placebo group (Vosskuhl et al., 2015). Further evidence for the causal role of both theta and gamma frequencies for working memory performance comes from an innovative approach stimulating with gamma waves that are nested onto a theta cycle (Alekseichuk et al., 2016). In a well controlled experiment, the authors stimulated gamma waves nested on a 6-Hz sine during a spatial working memory task. They report the strongest increase of performance for a gamma frequency of 80 Hz, while higher and lower gamma were not as successful. This finding illustrates the causal relevance of the specific frequency of the theta in combination with a gamma oscillation. In turn, it adds evidence to the theta-gamma coding theory of short-term memory and thus corroborates the idea that brain oscillations form a method of communication between brain regions.

### Phase

In the previous section, we already discussed that the minimum gap between two visual stimuli at which the participants were able to discriminate two flashes of light depends on their individual alpha peak frequency (Samaha and Postle, 2015). A similar effect has also been reported in relation to the phase of the alpha oscillation when two flashes of light were not only separated in time but also in space, resulting in either perceived simultaneity or succession of the light flashes (Varela et al., 1981). In this classic experiment, a higher probability of “simultaneity” ratings was reported if the light flashes were presented during the positive phase of an alpha oscillation as compared to the negative phase (Varela et al., 1981). Whereas earlier attempts to replicate the original results remained largely unsuccessfull (VanRullen and Koch, 2003), a more recent study claimed success when using a modified procedure of stimulus presentation (Milton and Pleydell-Pearce, 2016). The authors employed a lateralized presentation of the task using cued covert attention, which makes use of the more reliable detection of alpha phases during periods of increased alpha activity (Mathewson et al., 2009).

In another line of research, effects of alpha phase have been reported with regard to visual detection performance. When stimuli were presented near the detection threshold, the pre-stimulus phase of the alpha oscillation predicts whether the stimulus will be detected or not (Busch et al., 2009; Mathewson et al., 2009), that is, detection probability was high during the up-state and low during the down-state of alpha phase.

Despite an ever-increasing literature indicating a relationship of oscillatory phase to perception and cognition, there are only few studies using brain stimulation to test this hypothesis, probably because of the high chronological precision required for presenting stimuli at a specific phase (Ten Oever et al., 2016).

In the auditory domain, so far one study analyzed the causal role of the pre-stimulus alpha phase for stimulus detection (Neuling et al., 2012a). The authors presented sounds embedded in noise near the detection threshold and applied a 10 Hz oscillatory direct current stimulation (otDCS), a version of tACS with a direct current offset. In the positive half-wave of the otDCS, stimuli were perceived even at higher noise levels as compared to the negative half-wave (Neuling et al., 2012a), indicating a causal role of alpha-phase in auditory detection performance.

The relevance of oscillatory phase for auditory processing was also tested for the detection of a rhythmic sound stream in noise (Riecke et al., 2015). When tACS was applied at 4 Hz, a frequency identical to the rhythm of the presented soundstreams in that experiment, the phase of tACS determined the detection of the sound stream. When tACS and the sound were in phase with each other, detection was faster compared to the opposing condition (Riecke et al., 2015). Thus, tACS allows to facilitate or hinder the process of cortical synchronization to an incoming sound stream which in turn leads to changed detection rates.

Working memory, even more so than short-term memory, which has been discussed above, is a complex cognitive function that relies on an interplay between theta and gamma oscillations. It has been proposed that this interplay is realized by phase-amplitude coupling between theta and gamma waves. Thus, gamma waves are modulated in amplitude according to a specific phase of the underlying theta cycle (Lisman and Idiart, 1995; Axmacher et al., 2010). Accordingly, if tACS is applied with a suboptimal coupling of theta and gamma waves, performance should drop, whereas coupling at the optimal position should increase performance. This hypothesis was tested in an experiment that we discussed above (section “*Frequency*”) and which used a tACS signal of coupled theta and gamma waves (Alekseichuk et al., 2016). In their experiment, the authors did not only modulate the gamma frequency that was nested inside one theta cycle at 6 Hz, but also its position on the cycle. They found improved performance when gamma was nested in the peak of the theta wave as compared to a control condition where gamma was nested in the trough (Alekseichuk et al., 2016). This experiment clearly demonstrates a phase dependency between gamma amplitude and theta phase in working memory processes in the prefrontal cortex.

These behavioral effects which can be modulated by tACS at a specific frequency and phase demonstrate the relevance of the phase of a specific brain oscillation. With respect to brain functioning, these results indicate that brain oscillations serve as a process that structures information. On the one hand, incoming sensory information is actively gated by the phase of the alpha oscillation, on the other hand, processing of previously stored information is orchestrated by the phase of the theta oscillation.

### Phase Coherence

A special case of phase effects of brain oscillations is “phase coherence”—a state of fixed phase relationship between two cortical locations either between hemispheres or within one hemisphere. Interhemispheric phase coherence has been studied using a bistable apparent motion display that consists of two diagonally opposed pairs of point lights inducing the percept of either horizontal or vertical motion of the dots (Rose and Büchel, 2005). With central fixation of this display, stimuli appear on both sides of the visual hemifield and have to be integrated across hemispheres to generate perceived horizontal motion, whereas for perceived vertical motion, each hemisphere can process the stimuli independently (Chaudhuri and Glaser, 1991). In line with the documented role of gamma band (30–80 Hz) synchrony in object binding (Fries, 2005), interhemispheric coherence in the gamma band increased over occipito-parietal recording sites during perceived horizontal compared to vertical motion (Rose and Büchel, 2005).

Applying anti-phasic gamma tACS with 180° phase difference over motion-sensitive areas reduced the proportion of perceived horizontal motion, whereas in-phase stimulation (0° phase difference) did not affect the percept (Strüber et al., 2014). A comparison of inter-hemispheric coherence in the gamma-band in EEG data recorded before and after tACS revealed a coherence increase for the anti-phasic stimulation only. The authors pointed out, however, that their measure of coherence merely represents the relative stability of the gamma oscillations in both hemispheres, which might include a 180° phase difference (Strüber et al., 2014).

In a follow-up study using more focal high-density tACS (Helfrich et al., 2014a), the behavioral findings during anti-phasic stimulation were replicated, whereas the electrophysiological results showed a decrease of interhemispheric gamma coherence during anti-phasic tACS and an increase during in-phase stimulation. In addition, Helfrich et al. (2014a) found a correlation between the change of online-coherence and the change of the motion percept, providing strong evidence for online-entrainment of gamma oscillations.

Overall, these studies support the theory of binding by synchrony (Gray et al., 1989; Engel et al., 2001) in a unique way. The disruption of synchrony between brain areas over both hemispheres which is induced by tACS, proves that synchronous activity of the relevant areas form a coherent percept.

Effects of phase coherence on cognitive performance within one hemisphere have been studied in the context of theta oscillations and short-term memory (Polanía et al., 2012). These authors aimed at testing the causal role of theta coupling between frontal and parietal areas during visual memory matching. Theta oscillations serve as a channel of long-distance communication between two cortical areas, if the phase is coupled between the two areas. To test this hypothesis, tACS was administered in- vs. antiphasic to either synchronize or desynchronize the phase of the theta oscillation between parietal and frontal regions. The results showed that reaction times in response to a delayed match-to-sample task were reduced during synchronization induced by inphasic tACS and increased during desynchronization following antiphasic tACS (Polanía et al., 2012). This finding indicates a causal role of phase synchrony between two cortical areas for short-term memory. Unfortunately, direct physiological effects of tACS on oscillatory phase could not be analyzed because EEG was not recorded in the tACS experiment. The experiment was replicated, however, using four stimulation electrodes over both hemispheres (Alekseichuk et al., 2017). Additionally, it allowed to either synchronize or desynchronize frontal and parietal areas in both hemispheres instead of only the left hemisphere. Before and after stimulation, EEG was recorded. The study found a decrease in memory performance accompanied by an increase in reaction times in the desynchronized stimulation condition which is in line with Polanía et al. (2012). Additionally, the EEG data show a desynchronization of frontal and parietal EEG electrodes within both hemispheres after desynchronizing tACS which can explain the behavioral results.

Stimulation setups designed to either synchronize or desynchronize the oscillatory phase of a brain oscillation between two target areas have to be chosen such that the only difference between these two conditions is the phase of the electric fields underneath the stimulation electrodes. To ascribe an experimental effect only to the phase-difference between conditions, all other parameters, such as current flow direction, intensity at target locations, stimulated areas etc., must be kept constant (Saturnino et al., 2017). By modeling electric fields of electrode montages that had previously been reported, Saturnino et al. (2017) found that this goal was not reached for some of the montages. Especially a montage using two target and one “return” electrode in one condition and a pair of target electrodes in another condition (Polanía et al., 2012) led to differences in current flow direction between conditions. These additional un-intended manipulations can best be avoided using a combination of two concentric ring montages or 4 × 1 montages under certain conditions (Saturnino et al., 2017).

## Recent Developments

The field of (oscillatory) brain stimulation is still in its infancy. Since the first tACS study published 10 years ago (Antal et al., 2008), the method has been refined in many ways but there are still concerns about several issues. Current flow models allow for a more informed positioning of stimulation electrodes (Neuling et al., 2012b), changing the montage from conventional two conductive rubber pads to a 4 × 1 ring electrode configuration increases focality of stimulation (Bikson et al., 2009), improved machinery allows for more sophisticated stimulation signals (Dowsett and Herrmann, 2016; Witkowski et al., 2016). Nevertheless, there are many possible sources for varying outcomes of experiments with regard to study designs, stimulation protocols and not least individual differences in the responsiveness to tACS (Krause and Cohen Kadosh, 2014). To name but a few, the exact positioning of stimulation electrodes changes the current flow and thus influences the outcome of an experiment (Mehta et al., 2015), stimulation duration influences whether aftereffects can be observed or not (Strüber et al., 2015), internal factors like brain states (Neuling et al., 2013; Kasten et al., 2016) together with external factors like ambient illumination during stimulation (Stecher et al., 2017) have an impact on the efficacy of tACS. That is, alpha levels in dark surroundings are typically elevated due to fatigue increments during lengthy experiments, thus during darkness, tACS at a given intensity has not the same effect as in illuminated surroundings with lower alpha levels (Stecher et al., 2017). The list of parameters that are relevant for defining the efficacy of tACS is yet to be completed.

Such sources of variability might explain why the field suffers from a substantial amount of null-results and failed replications (Lafon et al., 2017). However, we believe that many of these null results will help the field to generate more refined hypotheses and, thereby, foster the investigation of mechanisms underlying tACS. They might also help to uncover even more factors that influence the outcome of tACS and to qualify or refute some previously established effects of tACS and assumptions about brain oscillations.

### Stimulating With Complex Waveforms

Usually, tACS is applied as a sinusoidal waveform which relates to the predominant way of treating brain oscillations as sinewaves, or combinations of sinewaves and of analyzing oscillatory time series data with sinusoidal models, such as FFT and wavelets. Despite its convenience, one has to be aware that this approach oversimplifies the nature of brain activity which takes rarely the form of a mathematical sine wave. Only because in EEG and MEG we observe reoccurring events at a certain frequency, which show up as a peak in the spectrum, this does not necessarily mean that this activity originated from sinusoidal activity. In this regard, it has been argued that taking into account non-sinusoidal shapes of waveforms might provide important new information about the computational properties of brain oscillations and alternative analysis methods for studying non-sinusoidal physiological waveforms have been suggested (Jones, 2016; Cole and Voytek, 2017).

In the field of tACS, there are two motivations to use waveforms different from a uniform sine wave. First, to avoid or reduce the electric stimulation artifact (Dowsett and Herrmann, 2016; Witkowski et al., 2016). When, for example, sawtooth waves are used, EEG activity obscured by the electric artifact can be restored more easily due to the linear increase of the current (Dowsett and Herrmann, 2016). In that study, artifact reduction was successful with both negative and positive ramps, but only the positive ramp wave resulted in the expected increase of alpha-activity (Dowsett and Herrmann, 2016). Since all other parameters of the waves were identical, the shape of the waveform is left as the only explanation for this result. A different approach to avoid the electric artifact is to use amplitude-modulated tACS (Witkowski et al., 2016; Minami and Amano, 2017). With this approach, stimulation consists of a carrier frequency outside the physiological range (e.g., 220 Hz) which is rhythmically modulated in amplitude at a physiological target frequency (e.g., 10 Hz). In theory, the envelope of the stimulation signal does not leave a trace in the spectrum, thus, the physiological activity at the envelope frequency can be analyzed after low-pass filtering of the signal (Witkowski et al., 2016; Minami and Amano, 2017). When applied in a setup including non-linearities, however, the measured signal contains an artifact at the modulation frequency (Minami and Amano, 2017; Kasten et al., 2018 (Supplements)).

The second motivation to use more complex waveforms instead of a simple sine wave is to test theories about frequency coupling or in cases when the exact waveform of the oscillation is thought to play a role for a specific cognitive function. A good example of this approach is given by the tACS study of Alekseichuk et al. (2016) on spatial working memory that has been mentioned already. In their experiment, the authors mimicked phase-coupled gamma oscillations on a theta wave with their stimulation signal. They found that the precise position of the gamma wave on the theta wave is relevant for working memory (Alekseichuk et al., 2016). These findings do not only substantiate the functional relevance of theta and gamma waves for cognition, but also support the notion of cross-frequency coupling as a mechanism of brain functioning in general.

Another example for making use of stimulation with more complex waveform shapes stems from the area of speech comprehension. When presented with speech sounds, the auditory cortex displays EEG activity in the theta band that appears as the envelope of the original speech signal (Luo and Poeppel, 2007; Abrams et al., 2008). This effect is thought to be relevant for speech comprehension (Doelling et al., 2014). Thus, tACS might foster speech intelligibility when applied as the exact speech envelope. This hypothesis has been recently tested by using such envelope-tACS with different delays between the stimulation and the auditory speech signal (Wilsch et al., 2018). The authors extracted the low frequency envelope of spoken sentences and used them as a stimulation signal for tACS. With this procedure, speech comprehension was improved for individually varying time delays between tACS signal and speech sound that had to be adapted (Wilsch et al., 2018). With a very similar approach, these findings were replicated (Riecke et al., 2018). In that study, the authors first used a stimulation frequency that is tailored towards the most prominent envelope frequency of each individual sentence presented. In a second experiment, they used a speech envelope as a stimulation signal comparable to Wilsch et al. (2018). With their work Riecke et al. (2018) extended previous findings to a two-talker situation. Additionally, tACS in the theta range (3.125 Hz) was found to affect the hemodynamic response in the auditory cortex evoked by speech stimuli (Zoefel et al., 2018). This finding supports the effectiveness of tACS on the auditory system in a different domain of measurement. Findings from this line of research might be relevant for the design of hearing aids in the future.

### Increasing Focality of Electrical Stimulation

Another field within electrical brain sitmulation which advances quickly is the development of stimulation protocols that are more focused than the widespread effects from stimulating with large (7 × 5 cm) conventional conductive rubber electrodes (Datta et al., 2009). Focality is improved by increasing the number of stimulation electrodes, arranged in a roughly circular fashion with one central electrode (Datta et al., 2009; Helfrich et al., 2014a). Currents flow between the central electrode connected to one “pole” and the surrounding electrodes connected to the other “pole” of the stimulator.

Simultaneous to the advancements in stimulation protocols, another key to reach more precise stimulation is to estimate the target of the electric currents more precisely (Miranda et al., 2003; Wagner et al., 2014). The electric fields inside the human head depend on the conductivity of the different tissues between the two (or more) electrodes. Individual anatomy, the exact electrode position and stimulation intensity influence which cortical brain area receives the strongest stimulation. Modelling the current flow inside the human head is a challenging task, since the optimal model depends on precise knowledge of individual anatomy and potentially individual conductivities. Early models of current flow used spherical head models with three compartments of different conductivities (Miranda et al., 2003). More realistic head models have been developed that use realistic shapes of different tissues, with up to six compartments plus white matter anisotropy derived from MRI measurements and a higher resolution of three-dimensional geometry of the head (Neuling et al., 2012b; Wagner et al., 2014). These studies present head models derived from MRI of an individual 26 year old male subject, and does not take into account inter-individual variability of the form, relative positioning and mass of the compartments. Also conductivity values are used from literature. The models could be improved even further by measuring anatomy and conductivities for each subject individually. Temporal changes of conductivities, e.g., due to sweating during the experiment, and even anatomical parameters (e.g., due to changing body positions) are still not taken into account.

A completely new account to stimulate in a more focused fashion is to use temporally interfering electric fields (Grossman et al., 2017). Using two distinct channels of stimulation, two slightly different frequencies are applied which both do not have a physiological representation. The difference between the two frequencies is the target frequency. In an area where the electric fields overlap, the sum of the two fields appears as an amplitude modulated signal at the modulation frequency of the difference between the two original frequencies (Grossman et al., 2017). The method however has only been tested in mice (Grossman et al., 2017) and awaits empirical validation in humans.

### Artifact Reduction

As mentioned above, the EEG signal is contaminated by a huge electric artifact when recorded during tACS. However, exactly this setup is needed to directly demonstrate that entrainment of brain oscillations was induced by tACS. Thus, artifact reduction is important for revealing online effects of tACS. Ideally, the artifact and physiological signals were separated from each other whenever oscillations at the stimulation frequency (or its harmonics) are to be analyzed. Apart from avoiding the problem by using a different stimulation signal (Dowsett and Herrmann, 2016; Witkowski et al., 2016; Minami and Amano, 2017), tACS artifact reduction methods have been inspired by notch-filtering (Voss et al., 2014) and by methods allowing for concurrent EEG-fMRI measurements (Helfrich et al., 2014b). With these approaches, one central question is still unresolved: Is it principally possible to reliably discern a small remaining artifact from entrained brain activity in EEG data? In order to be sure that an experimental effect is not merely a residual artifact, the size of the residual artifact has to be estimated and taken into account for statistical testing.

In MEG data, the artifact is potentially easier to tackle since the sensors and the stimulation electrodes are not electrically coupled. Using spatial filtering methods (Soekadar et al., 2013), the natural increase in alpha amplitude during closed eyes has been recovered from MEG data recorded during tACS at the IAF (Neuling et al., 2015). It is, however, an ongoing debate whether these methods can fully separate artifactual from entrained brain activity (Neuling et al., 2017; Noury and Siegel, 2018). The current state of the discussion is reviewed by Herrmann and Strüber (2017).

Besides the discussions about the validity of different methods to reduce the artifact, and the discussion about the size of the residual artifact, one way out might be to statistically contrast experimental conditions which both contain possible residuals of the artifact (Neuling et al., 2015; Kasten and Herrmann, 2017). Because the artifact may change over time, and respectively the residuals after correction, comparisons of data which is temporarily close together seems to be most valid (Kasten and Herrmann, 2017).

### Closed-Loop tACS

Brain states are often unstable. With the change of brain states, the oscillatory pattern of the electrophysiological signal changes over time. For example, the oscillatory frequency fluctuates, the amplitude of an oscillation changes and so on. In a traditional tACS experiment, individual stimulation parameters are defined at the beginning of the experiment (Vossen et al., 2015; Vosskuhl et al., 2015), thus ignoring the natural fluctuation of frequencies within a certain range (Vossen et al., 2015). In principle, tACS shows stronger effects, if the stimulation frequency is adapted to the internal frequency. With a fixed stimulation frequency, the internal and the stimulation frequency are not always matched—especially, when measuring over longer time-periods. To optimally adjust the stimulation frequency to the internal frequency, the internal frequency should be constantly monitored and the stimulation frequency must be constantly adapted respectively. Such a system would represent a closed loop control system. To date such a setup has not been realized on the basis of EEG recordings (Thut et al., 2017), probably due to the unresolved issue of the electric artifact during tACS stimulation. Alternatives to EEG as the basis for informed closed loop tACS may be the use of peripheral representations of cortical rhythms (Brittain et al., 2013), or the intermitted stimulation and EEG recordings (Lustenberger et al., 2016). Yet, we expect closed-loop tACS to lead to increased effect sizes and a more effective use of tACS in the future. With respect to clinical applications, closed-loop tACS offers the possibility to use brain stimulation more parsimoniously when stimulation is only required in malfunctioning brain states but not throughout normal brain functioning. The current state of closed loop applications of tACS and other NIBS techniques has been reviewed elsewhere (Bergmann et al., 2016; Karabanov et al., 2016; Thut et al., 2017).

## Outlook

With tACS in humans, researchers gained another level of control to study oscillatory processes in the brain. Even though the mechanism of tACS functioning are not yet fully understood, we expect the field of non-invasive brain stimulation to grow and increase its influence on neuropsychology in the near future. Once the methods are established and their mechanisms fully explained, they will fundamentally impact our understanding of brain functioning.

By the thoughtful application of alternating currents to the human scalp, oscillatory brain activity can be manipulated in its frequency, amplitude or phase. In turn, previously mostly correlative theories on the relevance of brain oscillations for human cognition can now be tested causally on samples of healthy humans. At the same time, the functional role of brain oscillations is established and a more holistic idea of brain functioning might emerge. If brain oscillations form the functional basis of brain functioning (Buzsáki, 2006), rhythmic brain stimulation forms an adequate tool to provide evidence.

### Long-Term Effects of tACS

With respect to research on the mechanisms of action behind tACS, we come to the conclusion that there is good progress in understanding the online effects of AC stimulation on cortical neurons and oscillations *in vitro*. Kar and Krekelberg (2014) reported modified neuronal adaptation as a consequence of tACS, which suggests that tACS can interfere with natural plasticity. However, with regard to the underlying mechanisms of long-term effects, a thorough investigation of tACS-induced plasticity in living animals or *in vitro* is still needed, as mentioned earlier (Strüber et al., 2015). If neural plasticity turns out to be the mechanism responsible for tACS aftereffects, the principles of plasticity can be utilized to establish long-lasting effects of tACS and tACS can be established as a therapeutic tool. The first steps towards tACS as a therapeutic tool for the treatment of neurophsychiatric disorders have already been taken. For example, tACS has been successfully applied to reduce symptoms of Parkinson’s disease (Brittain et al., 2013), ADHD in children (Munz et al., 2015) and other disorders related to malfunctioning brain oscillations, reviewed for example by Herrmann and Strüber (2017). Nevertheless, once long lasting effects of tACS can be robustly induced and fully explained, tACS can finally be eventually introduced as a possible treatment for conditions related to malfunctioning brain oscillations.

## Conclusion

Even though brain oscillations and other EEG or MEG signals have been described throughout the history of cognitive neuroscience, a detailed knowledge about the origin of that signal and how exactly it relates to brain function is still missing, as pointed out recently by Cohen (2017). This author claims that the usefulness of EEG signals in understanding brain functions depends on our knowledge about the interaction of cortical microcircuits. The current approach of correlational and for the most part macroscopic research is interpreted as “mapping the landscape” (Cohen, 2017), which does not sufficiently clarify the fundamental relationship between EEG-signals and cognitive processes, as regarded by the author.

This article is part of a Frontiers Research Topic “Paradigm shifts and innovations in Neuroscience.” We believe that tACS and other NIBS techniques will probably lead to major progress in human neuroscience. It has the potential to support currently correlational models of brain functioning with causal evidence. This probably does not constitute a paradigm shift in strict sense of Kuhn (1970) or a scientific revolution in the sense of Nickles (2017). However, NIBS will lead to refined or even profoundly new and more complete theories about brain functioning and thus represents at least a scientific progress as defined by Niiniluoto (2015).

## Author Contributions

JV, DS and CH conceptualized and wrote the article.

## Conflict of Interest Statement

CH has filed a patent application on brain stimulation and received honoraria as editor from Elsevier Publishers, Amsterdam. The other authors declare that the research was conducted in the absence of any commercial or financial relationships that could be construed as a potential conflict of interest.
